# Multiple green spectroscopic methods for erdosteine determination in bulk and dosage form with extensive greenness evaluation

**DOI:** 10.1038/s41598-023-45334-6

**Published:** 2023-10-25

**Authors:** Hadeel A. Khalil, Eman I. El-Kimary, Amira F. El-Yazbi, Tarek S. Belal

**Affiliations:** Pharmaceutical Analytical Chemistry Department, Faculty of Pharmacy, Alexandria University, 1 El Khartoum Square, Alexandria, 21521 Egypt

**Keywords:** Drug screening, Medicinal chemistry, Analytical chemistry

## Abstract

Four simple, sensitive, economical, and eco-friendly spectrophotometric and spectrofluorimetric methods for the assay of erdosteine (ERD) in bulk and dosage form have been developed and validated as per the current ICH guidelines. Method I involved the addition of the powerful oxidizing agent, potassium permanganate to ERD and measuring the oxidation product at 600 nm. Another oxidizing agent; ceric ammonium sulfate was used in Method II where ERD is oxidized resulting in a decline in the absorbance intensity of cerium (IV) ions, measured at 320 nm. Similarly, Method III employed the use of ceric ammonium sulfate, However, the fluorescence intensity of the resulting cerium (III) ions was recorded at λex/λem 255/355 nm, respectively. Whereas in Method IV, ERD was added to acriflavine leading to a proportional decrease in its native fluorescence. Various reaction conditions affecting the intensity of measurement were attentively investigated, optimized, and validated. All the suggested methods did not require any tedious extraction procedures nor organic solvents. The implementation of the proposed methods in ERD assay resulted in linear relationships between the measured signals and the corresponding concentrations of ERD in the range of 1–6, 0.1–1.0, 0.01–0.1, and 10–100 μg/mL with LOD values 0.179, 0.024, 0.0027 and, 3.2 μg/mL for methods I, II, III and IV respectively. The suggested methods were successfully applied to ERD analysis in pure form and in commercial capsules. Furthermore, the eco-friendliness of the proposed methods was thoroughly checked using various greenness testing tools. Lastly, this work, not only presents highly sensitive, green, mix-and-read methods for ERD determination, but also, describes the determination of ERD spectrofluorimetrically for the first time in the literature.

## Introduction

**Figure 1:**
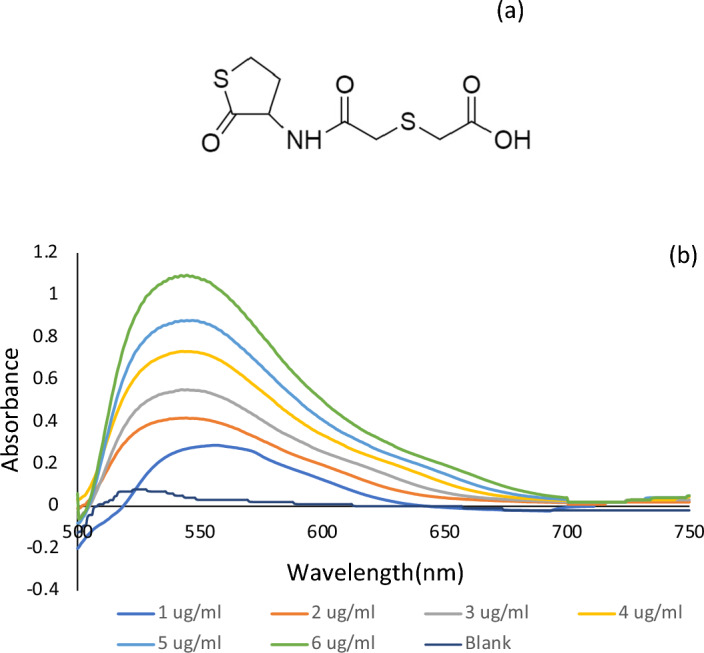
(**a**) Chemical structure of Erdosteine (ERD), (**b**) Absorption spectra of the reaction product of 0 (Blank),1, 2, 3, 4, 5 and 6 μg/mL of ERD with potassium permanganate in alkaline medium.

Erdosteine (ERD), is a potent expectorant that acts by regulating mucus production and reducing its viscosity in the respiratory tracts. Moreover, owing to its free radical scavenging action, ERD exhibits an ant-inflammatory role in the treatment of acute and chronic obstructive bronchitis. Chemically, the structure of erdosteine (2-[2-oxo-2-[(2-oxothiolan-3-yl)amino]ethyl]sulfanyl acetic acid) (Fig. 1a)^1–3^.

In general, several analytical techniques have been developed for the direct determination of ERD including UV spectrophotometric methods^3–7^. In addition, various chromatographic techniques were described in the literature for the quantitation of ERD in pharmaceutical dosage forms as well as biological samples. For instance, high performance liquid chromatography (HPLC) using UV^8–13^ and tandem mass detection^14^, high performance thin layer chromatography (HPTLC)^15^ and Ultra-High Performance Liquid Chromatography (UHPLC) with fluorimetric detection^16,17^. Validated methods for the simultaneous determination of cefixime and erdosteine in pharmaceutical dosage forms using UV spectrophotometry^18–20^, reverse phase HPLC^21^ and HPTLC^22,23^ have also been reported.

Meanwhile over the last years, the implementation of green analytical techniques has gained great importance in the analytical field^24^. This is attributed to the numerous benefits it offers over other commonly used quantification methods. These include its economic cost and the use of reduced volumes of aqueous buffers rather than hazardous solvents as acetonitrile, formaldehyde, and halogenated solvents and thus avoids the generation of harmful organic waste products^25,26^.

ERD chemical structure displays oxidizable groups, and hence potassium permanganate, KMnO_4_, (method I) and ceric ammonium sulfate, (NH_4_)_4_Ce(SO_4_)_4_.2H_2_O, (method II) were examined for their oxidizing effect on ERD. KMnO_4_ reacted with ERD in strong alkaline medium turning the violet color of potassium permanganate to green color of managante species^27–29^. Differently, ceric ammonium sulfate reaction was once employed in strong acidic medium (H_2_SO_4_) reducing the intensity of the yellow color of the ceric solution (method II) while in method III, the resulting fluorescent cerous ion from this reaction was quantitated^30^. In method IV, acriflavine has been used as a fluorescent quenching reagent for the determination of erdosteine. Acriflavine is a dye that has been previously used as a fluorogenic reagent for the quantitation of various drugs, such as ascorbic acid, ketoprofen, acemetacin and tranilust^31–35^. This chemical reaction relies on the ability of the drug to quench the acriflavine fluorescence quantitively allowing for accurate drug determination. At suitable pH, the carboxylic group in erdosteine could interact with acriflavine leading to a reduction of acriflavine native fluorescence. All the experimental conditions were assessed to ensure maximum sensitivity of the developed methods.

Moreover, the development of simple spectrophotometric and spectrofluorimetric methods for the analysis of ERD in its dosage forms would be advantageous to allow its sensitive, selective, fast, cost-effective green determination in quality control laboratories for content uniformity or batch-to-batch consistency testing using widely applicable techniques and instrumentation that are available in most laboratories.

To the best of our knowledge, the entire sum of UV spectrophotometric assays found in the literature employs direct measurement of ERD in dangerous chemical solvents. This work attempts to develop green, facile, and novel UV spectrophotometric and spectrofluorimetric methods for the accurate estimation of erdosteine (ERD) using simple derivatization reactions. Furthermore, the greenness of the developed methods is evaluated using various tools including; Analytical Eco-scale, Green Analytical Procedure Index (GAPI) and AGREE and is compared to other published techniques. The developed methods proved to be eco- friendly validated methods that can be routinely applied for the analysis of ERD in in bulk powder and pharmaceutical dosage forms (capsules).

## Experimental

### Instrumentation

Absorbance measurements were made using Specord S600 UV–VIS diode array spectrophotometer associated with 1-cm quartz cells and Winaspect software version 2.3 (Analytik Jena AG, Germany). Spectrofluorimetric measurements were carried out on Cary Eclipse Fluoresence Spectrophotometer from Agilent technologies, USA (model: G9800A), equipped with 1-cm quartz cell and 150-W xenon lamp. All pH measurements were recorded using (Crison Instruments SA, Barcelona, Spain). Effect of temperature was studied using a thermostatically controlled water bath, Daihan scientific, Korea.

### Materials and reagents

Erdosteine (purity > 99.85%) was a kind gift from Borg Pharmaceutical Industries (Borg El Arab New City –Alexandria – Egypt). All chemicals used were of analytical reagents grade. Methanol was obtained from Fisher Scientific, Loughborough, UK. Ceric ammonium sulfate (purity > 99%) was obtained from Nice Chemicals, Kerala, India. Acriflavine was purchased from Sigma-Aldrich, Germany. Boric acid was obtained from El-Gomhoreya, Pharmaceutical Chemicals Co., Egypt. Potassium permanganate glacial acetic acid, phosphoric acid and NaOH were obtained from El-Nasr Chemical Co., Egypt. H_2_SO_4_ (99.5%) was obtained from Loba Chemie Pvt.Ltd., India. Deionized water was used in the experiments.

### Preparation of reagents

Sodium hydroxide (0.5 M) solution was prepared by weighing 2 g of NaOH and dissolved in 100 mL deionized water. Sulfuric acid solution (2 M) was prepared by slowly adding a volume of 27 mL of concentrated H_2_SO_4_ to 250 mL deionized water. Britton–Robinson buffer (0.04 M in each of acetic, o-phosphoric and boric acids) adjusted to the required pH with 1 M sodium hydroxide solution was dissolved in deionized water. To prepare 0.005 M potassium permanganate, 80 mg of potassium permanganate was dissolved in 50 mL deionized water; eventually, the volume was made up to 100 mL using deionized water. On the other hand, ceric ammonium sulfate solution (2.3 × 10^–3^ M) was prepared by accurately weighting 37.5 mg of ceric ammonium sulfate and then dissolved in 10% H_2_SO_4_; eventually, the volume was made up to 250 mL using the same solvent. To prepare acriflavine aqueous solution (2 × 10^−5^ mol/L); 0.0013 g of acriflavine was dissolved in 250 mL deionized water.

### Pharmaceutical preparations

Erdolytic® capsules (labeled to contain 300 mg of erdosteine manufactured by Al Andalous for pharmaceutical industries, Egypt, and Mucotec 150® capsules (labeled to contain 150 mg erdosteine manufactured by Global Napi pharmaceuticals, Egypt) were obtained from the local market.

### Preparation of erdosteine (ERD) stock solutions

Twenty-five milligrams of ERD were quantitatively transferred into a 25 mL volumetric flask, dissolved in 10 mL deionized water and sonicated for 15 min and the resulting solution was diluted to the mark with deionized water to provide a stock standard solution (1000 μg/mL). Serial dilution with deionized water was used to get 0.1 mg/mL (100 μg/mL) (stock solution for methods I and II) and 0.01 mg/mL (10 μg/mL) (stock solution for method III). As for method IV, the stock standard solution (1000 μg/mL) was used. The prepared stock solutions were stored at 4 °C.

### General procedure

#### *Method I (Spectrophotometric determination by reaction with KMnO*_*4*_*)*

Two milliliters of KMnO_4_ were added to a set of 10 mL volumetric flasks. This was followed by the addition of 2 mL NaOH solution (0.5 M). To prepare the different concentrations of the calibration curve (1–6 μg/mL), appropriate aliquots of ERD stock solution (100 μg/mL) were added. The contents of the flasks were mixed thoroughly, and the reaction mixtures were left to stand at room temperature for 40 min. Deionized water was then used to complete the volume to the mark. At last, the absorbance readings were taken at λmax 600 nm against blank.

#### Method II (Spectrophotometric determination by reaction ceric ammonium sulfate)

To a set of 10 mL volumetric flasks, 1.6 mL of ceric ammonium sulfate were added followed by 0.5 mL of H_2_SO_4_ solution (2 M). To prepare the different concentrations of the calibration curve (0.1–1 μg/mL), appropriate aliquots of ERD stock solution (100 μg/mL) were added. The reaction mixture was then mixed and placed in a water bath set at 60 °C for 40 min with the flasks closed during heating. Deionized water was then used to complete the volume to the mark. The difference in absorbance (∆A) was recorded at 320 nm against blank.

#### Method III (Spectrofluorimetric determination by reaction ceric ammonium sulfate)

The same procedure used in (method II) was applied in (method III), however in this method ERD aliquots were taken from stock solution (10 μg/mL) to prepare various concentrations in the range of 0.01–0.1 μg/mL. The reaction mixture was then mixed and placed in a water bath set at 60 °C for 40 min with the flasks closed during heating. Deionized water was then used to complete the volume to the mark. The fluorescence readings corrected from blank reading (ΔF) were recorded at λex/λem 255/355 nm against blank.

#### Method IV (Reaction with Acriflavine)

Volumes of 2 mL of Acriflavine (2 × 10^–5^ mol/L) followed by 1.5 mL Britton–Robinson buffer solution (pH 5) were pipetted into a set of volumetric flasks (10 mL). Then appropriate aliquots of ERD solution (100 μg/mL) were added to yield concentration levels in the range of 10–100 μg/mL. The reaction mixture was left to stand for 7 min and then diluted to 10 mL using deionized water and mixed well at 25 ± 5°C. The difference in fluorescence intensity (ΔF) was measured against a blank with excitation at 265 nm and emission at 505 nm.

In method I, the calibration plots were constructed between the produced absorbance readings against the corresponding concentrations. While in method II, the calibration plots were constructed between the decrease in absorbance readings (∆A) against the corresponding concentrations. On the other hand, in method III the calibration plots were constructed between the increase in fluorescence intensities against the corresponding concentrations. Finally in method IV the calibration plots were constructed between the decrease in fluorescence readings (∆F) against the corresponding concentrations.

### Analysis methods for capsules

For each dosage form brand, capsule powder equivalent to 300 mg (Erdolytic®) or 150 mg (Mucotec 150®) ERD was added to 50 mL deionized water in 100 mL volumetric flasks. Then the flasks were ultrasonicated for 15 min. The obtained suspension was then filtered using Whatman® filter paper into 100 mL volumetric flasks. The obtained filtrate was completed to the mark with deionized water to obtain 3 and 1.5 mg/mL ERD for Erdolytic® and Mucotec® respectively. The stock solutions were further diluted to working solutions to obtain 1000 μg/mL (used as stock sample solution in method IV). The prepared stock sample solution (1000 μg/mL) was diluted with deionized water to obtain 100 μg/mL (this solution was used in methods I and II) and 10 μg/mL (this solution was used in method III). These solutions were used to get the concentrations in the used linearity ranges (Table 1). Ten milliliters volumetric flasks were used for the general procedures.

**Table 1:** Analytical parameters for the determination of ERD using the proposed methods.

Parameter reagent	KMnO_4_ (Method I)	Ce^4+^ ^(^Method II)	Ce^4+^ ^(^Method III)	Acriflavine (Method IV)
Wavelength(nm)	600	320	ex 255/em355	ex 256/em505
Apparent molar absorptivity (L.mol^-1^ cm^-1^)	40,837	265,015		
Concentration range (μg/mL)	1–6	0.1–1.0	0.01–0.10	10–100
Intercept(a)	0.0835	0.0595	−0.3929	−7.11
S_a_^a^	0.009	0.008	1.84	5.87
Slope(b)	0.164	1.06	2211	6.05
S_b_^b^	0.002	0.013	29.98	0.097
RSD% of the slope(S_b_%)	1.39	1.21	1.36	1.60
Correlation coefficient(r)	0.9996	0.9998	0.9997	0.9969
S_y/x_^c^	0.009	0.009	2.19	7.54
F^d^	5157	6816	5437	646
Significance F	2.25 × 10^–7^	3.92 × 10^–6^	5.50 × 10^–6^	1.42 × 10^–5^
LOD(μg/mL)^e^	0.179	0.024	0.0027	3.20
LOQ(μg/mL)^f^	0.542	0.074	0.008	9.69

^a^Standard deviation of the intercept.

^b^Standard deviation of the slope.

^c^Standard deviation of residuals.

^d^Variance ratio, equals the mean of squares due to regression divided by the mean of squares about regression (due to residuals).

^e^Limit of detection.

^f^Limit of quantification.

## Results and discussion

### Spectral characteristics of the proposed spectroscopic procedures

In this work, four chemical derivatization procedures were validated to allow for ERD assay under green conditions. Looking at ERD structure (Fig. 1a), some functional groups are noted to be liable to oxidation, such as the two sulfur groups.

In method I, pilot tests have shown that ERD is readily oxidized by KMnO_4_ in presence of alkaline medium (NaOH) at room temperature with the generation of green manganate ions which absorbs at λmax 600 nm. In this reaction an obvious bathochromic shift is demonstrated where permanganate species absorb maximally at 525 nm. The concentration of ERD is directly proportional to the intensity of the produced green color. Figure 1b demonstrates the absorbance curves obtained for different ERD concentrations after treatment with KMnO_4_ in alkaline medium.

**Figure 2:**
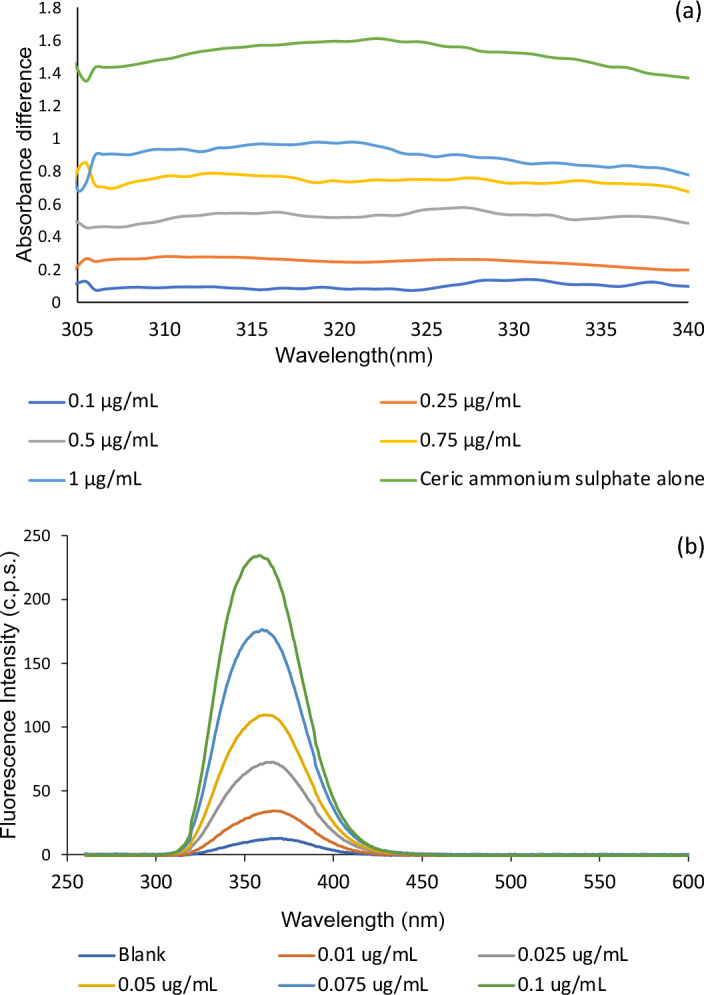
(**a**) Absorption spectra of the reaction product of ceric ammonium sulphate alone and with 0.1, 0.25, 0.5, 0.75, and 1 μg/mL of ERD, (**b**) Fluorescence emission spectra of the reaction product of 0, 0.01, 0.025, 0.05, 0.075, and 0.1 μg/mL ERD with ceric ammonium sulfate.

While in method II, ERD reacts with the strong oxidizing agent, ceric ammonium sulfate in acidic medium (H_2_SO_4_) leading to the reduction of yellow cerium (IV) ions into colorless cerium (III) ions. This is accompanied by an overall decline in the intensity of the yellow color measured at 320 nm. The reaction was investigated at different temperatures, and it was found that increasing temperature up to 60°C has remarkably enhanced the reaction sensitivity and hence, the reaction was performed at 60°C. The decrease in color was used for the spectrophotometric determination of concentration of ERD. Figure 2a illustrates the absorbance of different concentrations of ERD after reaction with ceric reagent in acidic medium at 60°C.

On the other hand, method III was developed in a similar context to method II where ceric ammonium sulfate in acidic medium (H_2_SO_4_) was implemented in the determination of ERD. However, instead of measuring the decrease in cerium (IV) ions absorbance intensity, the fluorescence intensity of cerium (III) ions was recorded at λex/λem 255/355 nm, respectively. Figure 2b shows the fluorescence emission spectra of different concentrations of ERD after reaction with ceric reagent in acidic medium at 60°C.

**Figure 3:**
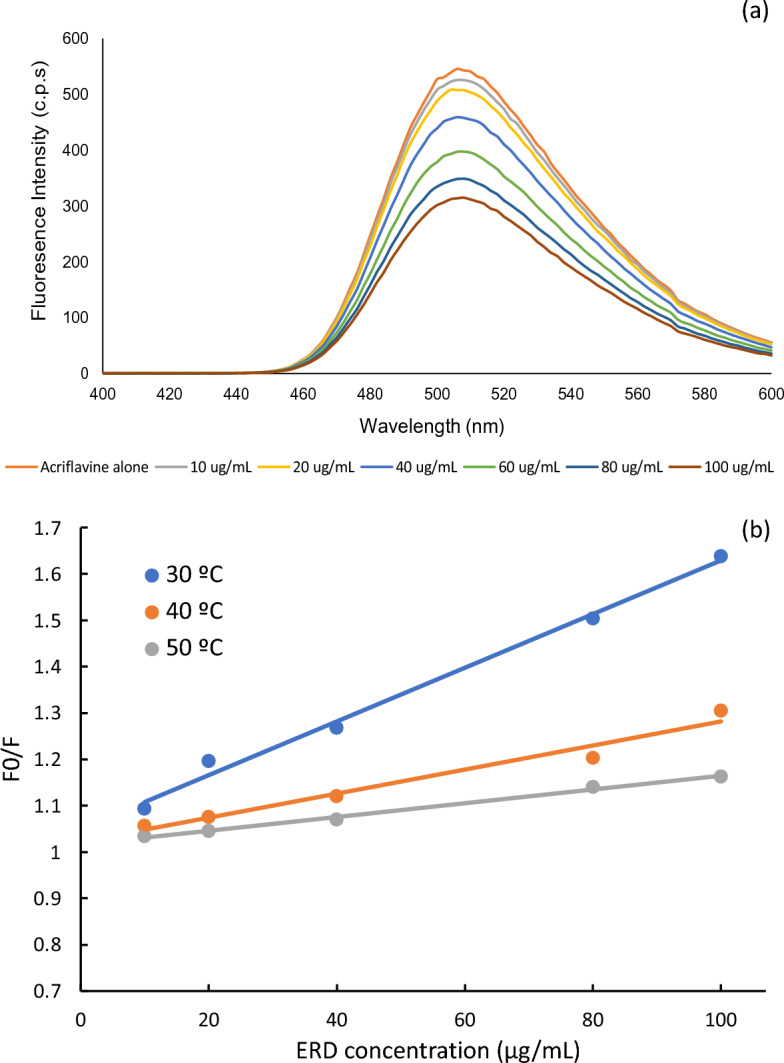
(**a**) Fluorescence Emission spectra of the reaction product of 0, 10, 20, 30, 40, 60, 80, and 100 μg/mL ERD with acriflavine reagent and (**b**) Stern–Volmer plot of the reaction of ERD with acriflavine at different temperatures (30 °C, 35 °C, 40 °C).

Finally, in method IV, acriflavine, a reagent with high native fluorescence at λex/λem 265/505 nm, was used for the estimation of ERD concentration. The addition of serial concentrations of ERD to a solution containing acriflavine (2 × 10^−5^ mol/L), under optimum conditions, led to a proportional reduction in the fluorescence intensity (Fig. 3a), This is attributed to the formation of an ion‐associated complex during quenching of the acriflavine native fluorescence. Supplementary Fig. S1 (in supplementary file) shows the excitation-emission spectra of the reaction product formed between acriflavine and ERD.

**Figure 4:**
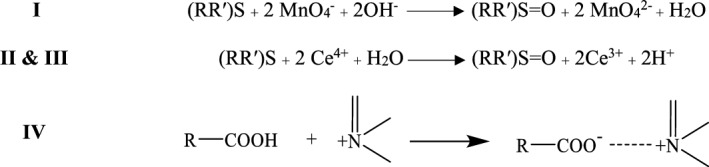
Mechanisms for the reactions between ERD and the different reagents; (I) KMnO_4_, (II and III) Ce^4+^, (IV) Acriflavine.

Job’s method of continuous variation was employed to understand the stoichiometry of the reactions (I-IV). Regarding methods I, II, and III, the ratio was found to be 1:2 (ERD: Reagent) considering the fact that ERD has 2 sulfur moieties that are liable to oxidation. As for method IV, the proposed mechanism for reaction IV was the formation of an ion associated complex between ERD and acriflavine in the ratio 1:1. This can be explained by the positive charge on nitrogen atom in acriflavine would form the ion-pair complex with the carboxylate anion in ERD through electrostatic forces of attraction. The suggested mechanisms for the proposed reactions are presented in Fig. 4.

In method IV quenching may occur due to various reasons, such as excited state reactions, energy transfer, complex-formation and collisional quenching. Dynamic quenching is due to interaction of the excited state of the fluorophore with the quencher which results in radiationless deactivation of the fluorophore to the ground state. While static quenching occurs when the fluorophore and the quencher form a complex in the ground state, i.e. before excitation occurs^31–35^. It is possible for the quenching to occur through different mechanisms; such as emission group destruction, molecular rearrangements, energy/electron transfer…etc^31–35^. In order to clarify the potential quenching mechanism, the following Stern–Volmer Equation was followed

$${\text{F}}_{0} /{\text{ F }} = { 1 } + {\text{ K}}_{{{\text{sv}}}} \left[ {{\text{ERD}}} \right]$$

where F_0_ and F stands for the fluorescence intensities in the absence and presence of drug, respectively, K_sv_ refers to the Stern–Volmer quenching constant, and [ERD] is the drug molar concentration.

To distinguish between static quenching and dynamic quenching, the temperature dependency of the Stern–Volmer plot was explored. K_sv_ value increases with increasing the temperature in case of dynamic quenching while, it decreases with increasing the temperature in case of static quenching. Dynamic quenching takes place at higher temperatures as diffusion occurs more rapidly. On the other hand, static quenching is generally less effective at higher temperatures due to the dissociation of weakly bound complexes. For this purpose, five different ERD concentrations were measured at several temperatures (30, 40 and 50 °C) and the obtained Stern–Volmer plots were compared (Fig. 3b). The values of K_sv_ were calculated and were found to decrease with the increase of temperature (0.0058, 0.0026 and 0.0015 mL· μg^−1^, respectively). As a result, it was concluded that, static quenching mechanism is responsible for the acriflavine quenching in presence of the studied drug.

### Reaction conditions optimization

**Figure 5:**
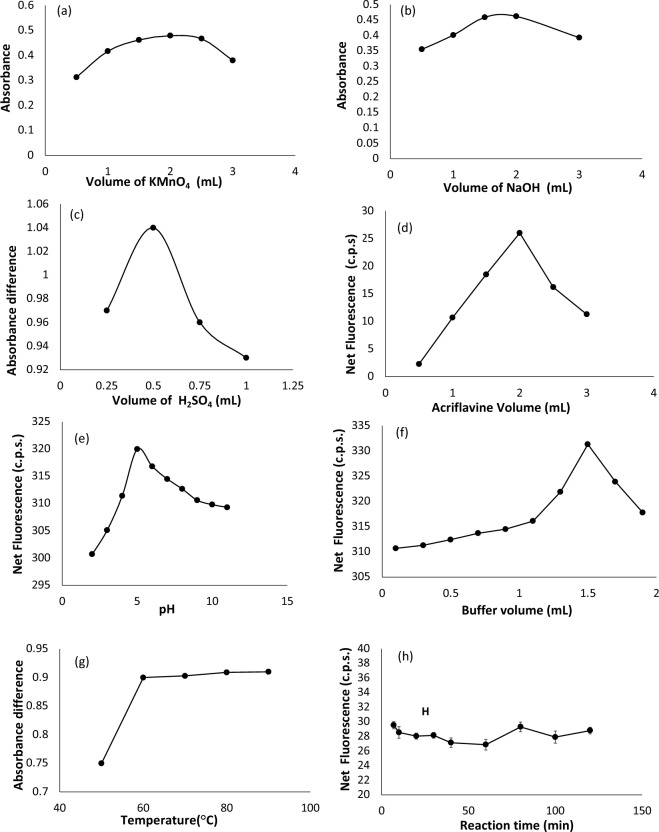
(**a**) Effect of different KMnO_4_ volumes on the reaction product of method I, (**b**) Effect of different NaOH volumes on the reaction product of method I, (**c**) Effect of different H_2_SO_4_ volumes on the reaction product of method II, (**d**) Effect of different acriflavine volumes on the reaction product of method IV, (**e**) Effect of pH on the reaction product of method IV, (**f**) Effect of different buffer volumes on the reaction product of method IV, (**g**) Effect of temperature on the reaction product of method IV, (**h**) Effect of reaction time on the reaction product of method IV.

The reaction variables affecting the formation of the measured reaction products were investigated to optimize the obtained results and ensure that the developed ERD assay methods are reliable. Each investigated factor was altered while others were fixed. The optimum conditions are listed in (Supplementary Table S1 in the supplementary file). The concentration effect of the reagent was observed in terms of reagent volume (0.005 M KMnO_4_, (2.3 × 10^–3^ M) ceric ammonium sulfate and (2 × 10^–5^ M) acriflavine). Figure 5 demonstrates the results. According to the obtained data, 2 mL KMnO_4_ were adequate to produce the highest absorbance reading. The volume of KMnO_4_ was increased above 2 mL, yet no increase in the color intensity was noted (Fig. 5a).

Moreover, the alkalinity required to complete the oxidation reaction was provided by NaOH solution (0.50 M). To monitor the effect of NaOH concentration, the reaction mixtures were prepared in different volumes of NaOH (0.5, 1.0, 1.5, 2.0 and 2.5 mL). The color intensity of the green product was enhanced by increasing the NaOH solution volume. However, exceeding 2 mL of NaOH led to a decrease in the absorbance readings. The effect of NaOH concentration on the obtained color product is shown in (Fig. 5b).

As for method II, which relies on measurement of the decrease in the absorbance of ceric ammonium sulfate, the maximum absorbance of 1.5 was obtained using 1.6 mL ceric ammonium sulfate (same volume was used in method III). Additionally, the acidity source was H_2_SO_4_ (2 M) solution. Different volumes of H_2_SO_4_ solution were used to prepare the reaction mixtures (0.25, 0.50, 0.75 and 1.00 mL) followed by measurement of absorbance of ceric reagent in presence and absence of ERD. As the volume of added H_2_SO_4_ solution was increased as the suppressive effect of ERD on ceric absorbance is increased. However, further increase of H_2_SO_4_ volume above 0.5 mL has reduced the suppressive effect and hence, 0.5 mL H_2_SO_4_ was chosen as the optimum volume. The impact of increasing H_2_SO_4_ concentration on absorbance difference (∆A) upon addition of ERD to ceric reagent is illustrated in (Fig. 5c).

In method IV, acriflavine volume was set at 2 mL as the greatest quenching effect was achieved using this volume (Fig. 5d). Moreover, the optimum pH of Britton–Robinson buffer was studied by altering the pH (2–10) and observing for its impact on the quenching of acriflavine fluorescence. Optimum quenching of fluorescence intensity was obtained when Britton–Robinson buffer solution pH 5 was used (Fig. 5e). Additionally, optimum buffer volumes were investigated. Different volumes (0.2, 0.4, 0.6, 0.8, 1, 1.2, 1.4, 1.6 or 1.8 mL) of Britton–Robinson buffer were added to ERD (Fig. 5f). The greatest fluorescence intensity resulted when 1.5 mL of the buffer was used. Hence, 1.5 mL of Britton–Robinson buffer solution pH 5 was selected throughout the study.

Reviewing the literature for method I, several studies have reported that the oxidation of drugs with permanganate in alkaline media are better done at room temperature rather than elevated temperatures^36–40^. This is due to the negative influence of high temperatures on the reproducibility of the reaction. Therefore, this experiment was conducted at room temperature. In method II and III, heat was essential for the complete oxidation of ERD reaction with ceric. In a thermostatically controlled water bath, the temperature of the reaction mixture was altered (50–90°C) to investigate the effect of heating on the oxidation of ERD by ceric. It was clear that increasing the temperature, increases the suppressive effect of ERD on ceric absorbance until it reaches a maximum at 60°C. Hence, 60°C was selected as the optimum temperature at which the reaction should be performed (Fig. 5g). As for method IV, heating the reaction mixture did not enhance the fluorescence signal measurement. Thus, the reaction was performed at ambient temperature.

The effect of reaction time on the measured signal was studied at room temperature (25 ± 5°C) for methods I and IV after adequate mixing of the reaction mixture. In method I, the reaction mixture was left to stand for up to 70 min and every 10 min the signal was measured. As the incubation period increased, the absorbance reading improved until it reached a plateau at 40 min. For method IV, as shown in (Fig. 5h), the incubation of the reaction mixture for longer periods than 7 min had no noticeable effect on the fluorescence with no significant changes for up to 120 min. Therefore, quenching of the acriflavine fluorescence signal was determined after 7 min. For method II, the reaction mixture was heated at 60°C for up to 70 min and the difference in absorbance (∆A) was determined every 10 min. Allowing the solutions to stand for 40 min has resulted in the greatest sensitivity of the measurements. Accordingly, in method III, measurement of the fluorescence signal was performed after 40 min. Figure 5h illustrates the effect of time on the measured signals.

The greenness of the proposed methods is evident by the use of water as the solvent throughout the experiments. This provides an excellent advantage over other analytical methods considering the reduced economic cost and the avoidance of harmful organic solvents usage. Supplementary Table S1 (in supplementary file) summarizes the optimized experimental conditions for the four methods.

### Validation of the proposed methods

In order to validate the investigated methods, the validation guidelines of analytical procedures (Q2R1) of the International Conference on Harmonization (ICH) were followed^41^.To investigate the linearity of the suggested methods, series of different concentrations of ERD were prepared. The least-squares treatment of the signal response data versus the corresponding concentrations was used to generate the linear regression equations. The obtained readings were directly proportional to corresponding concentrations of ERD. Regression analysis for the calibration curves of the proposed methods showed good linear relationship over the estimated concentration ranges. The RSD% of slope values (Sb%) for all four methods did not exceed 2% indicating good precision. The correlation coefficients for the methods (r) were acceptable for all the methods within the range from 0.997 to 0.9998. The limit of detection (LOD) and the limit of quantitation (LOQ) were calculated according to the mathematical equations in the ICH guidelines; LOD = 3.3σ / S, and LOQ = 10σ / S, where σ is the standard deviation of the response and S is the slope of the calibration curve. The highest sensitivity was obtained using method III (ceric spectrofluorimetry) reaching a limit of detection (LOD) of 0.0027 μg/mL. All the obtained data are listed in Table 1.

The accuracy, intra-and interday precision for the proposed methods were evaluated at three different concentrations levels of ERD (Table 2). The accuracy (Er %) and precision (RSD %) of the proposed methods were verified where most of the values were less than 2%.

**Table 2:** Intraday and interday precision and accuracy for the determination of ERD in bulk form using the proposed methods.

Method		Nominal concentration (μg/mL)	Mean ± SD^a^ (μg/mL)	RSD%^b^	E_r_%^c^
KMnO_4_ (Method I)	Intraday	2	2.01 ± 0.02	1.00	0.50
		4	3.97 ± 0.06	1.51	−0.75
		6	5.98 ± 0.03	0.50	−0.33
	Interday	2	2.03 ± 0.02	0.99	1.50
		4	3.94 ± 0.03	0.76	−1.50
		6	5.96 ± 0.05	0.84	−0.67
Ce^4+^ Spectrophotometry (Method II)	Intraday	0.5	0.508 ± 0.005	0.98	1.60
		0.75	0.750 ± 0.014	1.87	0.00
		1	1.02 ± 0.014	1.37	2.00
	Interday	0.5	0.506 ± 0.003	0.59	1.20
		0.75	0.749 ± 0.006	0.80	−0.13
		1	1.000 ± 0.016	1.60	0.00
Ce^4+^ Spectrofluorimetry (Method III)	Intraday	0.025	0.0254 ± 0.0004	1.58	1.60
		0.05	0.0505 ± 0.0006	1.19	1.00
		0.075	0.076 ± 0.0008	1.05	1.33
	Interday	0.025	0.0251 ± 0.0003	1.20	0.40
		0.05	0.0508 ± 0.0003	0.59	1.60
		0.075	0.075 ± 0.001	1.33	0.00
Acriflavine(Method IV)	Intraday	20	20.1 ± 0.34	1.69	0.50
		40	40.4 ± 0.68	1.68	1.00
		60	60.1 ± 0.02	0.03	0.17
	Interday	20	20.4 ± 0.30	1.47	2.00
		40	40.1 ± 0.86	2.14	0.25
		60	60.4 ± 0.24	0.40	0.67

^a^Mean ± SD for three determinations. ^b^% Relative standard deviation. ^c^% Relative error.

The robustness of the developed methods was studied by making small deliberate variations in the experimental conditions and monitoring its impact on the obtained results. These included alterations in the concentration of reagents, volume of alkali (NaOH) for method I, volume of acid (H_2_SO_4_) for method II, pH and volume of buffer (Britton robbinson) for method IV and temperature for method II, reaction times and wavelengths on the performance of the suggested methods. These experiments were performed by keeping all parameters constant except the parameter investigated and for each alteration the percentage recovery was calculated. None of these alterations had a noticeable effect on the performance of the investigated methods. The recoveries ranged from 98.2 to 100.9% with SD values ranging from 0.01 to 0.28 (Table 3). In addition, the relative standard deviations were less than 1.87, 1.02, 1.75 and 1.40 for method I, II, III and IV respectively. This confirms the reliability and robustness in routine application of the proposed methods in the analysis of ERD.

**Table 3:** Evaluation of the robustness of the proposed spectrophotometric methods for the determination of ERD by various reagents.

	Parameters	Mean rec. % ± SD^a^	RSD %^b^
Method I	KMnO_4_ volume ± 0.2 mL	100.41 ± 1.86	1.85
	NaOH volume ± 0.2 mL	98.62 ± 1.43	1.45
	Reaction time ± 5 min	98.31 ± 1.87	1.90
	Wavelength ± 2 nm	98.23 ± 0.83	0.85
Method II	Ce^4+^ volume ± 0.1 mL	99.59 ± 0.83	0.83
	H_2_SO_4_ volume ± 0.05 mL	98.96 ± 0.80	0.81
	Reaction time ± 5 min	98.21 ± 0.77	0.78
	Wavelength ± 2 nm	99.00 ± 0.13	0.13
Method III	Wavelength ± 2 nm	98.78 ± 1.20	1.22
Method IV	Acriflavine volume ± 0.2 mL	100.89 ± 1.20	1.19
	Buffer volume ± 0.2 mL	100.25 ± 1.41	1.41
	Buffer pH ± 2 units	100.46 ± 1.76	1.75
	Reaction time ± 2 min	101.59 ± 0.68	0.67
	Wavelength ± 2 nm	101.59 ± 0.24	0.24

^a^Mean percentage recovery for three determinations.

^b^% relative standard deviation.

Stock standard solutions of ERD were stable for 2 weeks when stored in the refrigerator at 4 ºC. In addition, the absorbance/fluorescence readings of obtained color solutions remained stable for one hour at room temperature.

### Assay of ERD in commercial dosage form

The proposed methods were successfully applied to ERD analysis in Erdolytic® and Mucotec® capsules labeled to contain 300 mg and 150 mg erdosteine (ERD) per capsule respectively. The results obtained by the recommended procedures are summarized in Table 4. The obtained data confirmed good accuracy and precision of the developed methods. One-way analysis of variance (ANOVA) was used to test if there was a statistically significant difference between the developed methods. Since the calculated p values were found to be 0.16 and 0.11 for the assayed dosage forms (more than 0.05) and the F value (1.95 and 2.23) were less than F critical (3.24), the null hypothesis was accepted. This proves a good agreement between the suggested methods. The obtained data proved that the suggested methods can be applied to ERD analysis in its capsules.

**Table 4:** Assay results for the determination of ERD using the proposed methods.

	Method I	Method II	Method III	Method IV
**Erdolytic® capsules**				
% Mean Recovery ± SD^a^	99.7 ± 0.46	99.8 ± 0.42	99.8 ± 0.42	100.3 ± 0.46
RSD%^b^	0.46	0.42	0.42	0.46
Er %^c^	−0.3	−0.2	−0.2	0.3
Anova (Single factor)	Alpha value	*p*-value	F Statistic	F Critical
	0.05	0.16	1.95	3.24
**Mucotec® capsules**				
% Mean Recovery ± SD^a^	99.9 ± 0.54	100.6 ± 1.09	100.6 ± 0.87	99.5 ± 1.45
RSD%^b^	0.53	1.09	0.87	1.45
Er %^c^	−0.1	0.6	0.6	-0.5
Anova (Single factor)	Alpha value	*p*-value	F Statistic	F Critical
	0.05	0.11	2.23	3.24

^a^Mean % recovery ± SD for five determinations.

^b^% relative standard deviation.

^c^% Relative error.

### Greenness assessment of the proposed methods

Green analytical chemistry (GAC) protocols were established to ameliorate the ecological burden and health hazards posed by the application of analytical procedures. A wide variety of GAC metrics have been employed to assess the greenness of analytical procedures. The greater the number of the GAC metrics used, the more comprehensive is the information provided about the analytical procedure, and the clearer is the green assessment profile. The proposed methods were evaluated using three different GAC metrics to confirm their greenness namely; General Analytical Procedure Index (GAPI), Analytical Eco scale, and Analytical Greenness (AGREE). Implementation of these effective tools have been demonstrated in several recent publications describing green spectrophotometric and spectrofluorimetric assay methods^24,25,42–44^.

The General Analytical Procedure Index (GAPI) was popularized in 2018 by Poltka-Wasylka^45^. The GAPI illustrative tool consists of a certain figure with five pentagrams investigating the greenness of the procedure according to 15 parameters through the traffic light colors (red, yellow and green) symbolizing high, medium to low impact, respectively. The entire analytical procedure is thoroughly rated by the GAPI metric system by assessing the sample preparation, reagents and solvents, instrumentation used where the circle in the middle depicts whether the method can be applied for qualification and quantification purposes. The extensive and easily perceivable information provided by the GAPI assessment made it a convenient tool for potential users/readers searching for greener analytical techniques. The proposed methods comprise a minimal number of steps all of which were performed under normal conditions, using no organic solvents and consuming the least possible amount of energy. Table 5 displays the GAPI models for the developed methods together. The GAPI pictogram colors of all four methods ranges from major green to minor yellow pentagrams with only two red ones depicting a high degree of greenness in the suggested methods compared to three red and lower frequency of green pentagrams in the reported spectrophotometric method^7^.

**Table 5:** Greenness evaluation of the proposed methods using different metrics.

Methods	GAPI	AGREE	Analytical Eco-Scale
I			87
II			94
III			94
IV			89

In 2020, a novel, full-scale approach has been introduced by Pena-Peveira^46^, the Analytical Greenness (AGREE). This assessment is based on the twelve principles of green analytical chemistry (GAC) and can be feasibly performed through a free access calculator. The results are concluded via a circular pictogram displaying the score (a fraction of unity from zero to one) in the center and is divided into twelve sectors. The degree of agreement with the GAC principles is represented by giving a certain color to each one of the sectors. Overall, AGREE metric is regarded as a broad spectrum, straight forward, rapid, and easily accessible method for greenness profile assessment. Implementing AGREE in assessing the developed methods has confirmed their greenness by the high scores obtained reaching 0.76 by method IV (Spectrofluorimetric method using acriflavine) Table 5. Whereas, the previously reported spectrophotometric methods 7 have scored values as low as 0.61 owing to the use of organic solvents such as acetonitrile, methanol and reagents such as chloranil and 7,7,8,8-tetracyanoquinodimethane (TCNQ).

Another tool for the verification of greenness of analytical procedures is the Analytical eco-scale. This system relies mainly on the quantity, any potential safety and/or health hazards of the chemical reagents and the energy consumed during application of the investigated procedure^47–49^. Analytical eco- scale is determined by subtracting the total penalty points from 100. Greater scores imply improved greenness in the suggested methods. The greenness of the proposed methods over the other reported spectrophotometric method^7^ is immediately concluded by considering the greater analytical eco scales reached (up to 94) whereas the reported method has obtained a score of 85 only Table 5. This may be attributed to the use of highly hazardous solvents such as acetonitrile, and methanol. This provides clear evidence that our methods have enhanced greenness over the reported method. Additionally, our work presents four different uncomplicated methods with improved sensitivities and wider linearity ranges in comparison with the previously reported method. The penalty points used to calculate the analytical eco scale for each method is provided in Table 6.

**Table 6:** The penalty points for the determination of ERD using the different proposed methods.

Analytical eco-scale	Method I	Method II	Method III	Method IV
Reagents/instruments	Penalty Points (PP)			
Potassium permanganate	8			
Ceric ammonium sulphate		1	1	
Acriflavine				4
Sodium hydroxide	2			
Sulphuric acid		2	2	
Britton robinson buffer				4
Energy				
UV Spectrophotometer	0	0		
Spectrofluorimeter			0	0
Occupational hazard	0	0	0	0
Waste 10 mL	3	3	3	3
Total penalty points	13	6	6	11
Analytical eco-scale total score	87	94	94	89

### Comparison of the developed methods with reported methods

Various methods were reported for the spectrophotometric determination of ERD (2–21). Our work presents spectrophotometric methods for the determination of ERD in the visible region. This is considered advantageous to other published spectrophotometric methods as it reduces possible interferences from any UV absorbing excipients present in the dosage form. Applying the developed methods, lower linearity ranges were reached than those of the reported method^7^ (10–500 μg/mL and 20–600 μg/mL) which indicates the greater sensitivity of our work. In particular, method III (Spectrofluorimetric reaction with ceric) with a LOD of 2.7 ng/mL providing an unparalleled sensitivity compared to the other reported method^7^. Moreover, to the best of our knowledge, no spectrofluorimetric methods have been published for ERD so far. Only one report described UPLC analysis of the drug with fluorimetric detection based on precolumn derivatization with 4‐bromomethyl‐7‐methoxycoumarin 17. This makes our study the first simple spectrofluorimetric determination method for ERD.

On the other hand, several chromatographic methods have been reported for the quantification of ERD in bulk, dosage forms and plasma, yet our work stands out in comparison with those methods regarding both sensitivity and greenness of the employed procedures. Table 7 summarizes the different reported methods used for ERD estimation and highlights the differences in sensitivity, minimal energy consumption, simplicity of procedure, and greenness of the solvents used in our work compared to other published methods. With comparable detection limits around in the range from 0.0027 to 3.200 μg/mL and analytical eco scale score of 94 (greater than all the values scored by the reported chromatographic methods), the superiority of our methods over previously published procedures is evident. In addition, the suggested procedures involves fast and simple steps that does not require expensive instrumentation, complicated extraction, or exhausting multi-step analytical schemes. The greenness of the suggested methods is testified using aqueous solutions of the reagents in the developed methods compared to other hazardous solvents used in the previously published methods such as methanol and acetonitrile. This promotes the proposed method to be used for high-through-put quality control analysis in various laboratories that can be further extended to various bioavailability studies.

**Table 7:** Comparison between the proposed methods and other reported methods used for the assay of erdosteine (ERD).

Method	Reference	Detection	Experimental conditions	Linearity range	LOD	LOQ	Ecoscale Score
Spectrophotometry I	Proposed Methods	UV/Visible	-ERD dissolved in Water -KMnO_4_ -NaOH -λ_max_ 600 nm	1–6 μg/mL	0.179 μg/mL	0.542 μg/mL	87
Spectrophotometry II		UV/Visible	-ERD dissolved in Water -Ceric ammonium sulphate -H_2_SO_4_ -λ_max_320 nm	0.1–1 μg/mL	0.024 μg/mL	0.074 μg/mL	94
Spectrofluorimetry III		Fluoresence	-ERD dissolved in Water -Ceric ammonium sulphate -H_2_SO_4_ -λex 255/λem355	0.01–0.1 μg/mL	0.0027 μg/mL	0.008 μg/mL	94
Spectrofluorimetry IV		Fluoresence	-ERD dissolved in Water -Acriflavine -BR-Buffer -λex 256/λem505	10–100 μg/mL	3.20 μg/mL	9.69 μg/mL	89
Spectrophotometry	^7^	UV/Visible	-Chloranil -λ_max_ 454 nm	10–500 μg/mL	–	10 μg/mL	85
Spectrophotometry	^7^	UV/Visible	-TCNQ - λ_max_ 843 nm	20–600 μg/mL	–	20 μg/mL	85
HPLC	^8^	UV	*SP:ODS column *MP: Water– acetonitrile, 0.1% TFA	0.02–6 μg/mL	–	0.02 μg/mL	86
HPLC	^11^	UV	SP: C18 (4.6 × 250 mm) column MP: Acetonitrile in phosphate-heptane sulfonate buffer (5:95)	0.5–8 μg/mL	–	0.5 μg/mL	82
HPTLC	^15^	Densitometric	SP:Aluminium-packed silica gel 60 F 254 plates MP: Toluene:methanol:acetone:ammonia (3.5:5:2.5:0.05)	30–1000 ng	–	30 ng	70
UPLC	^17^	Fluoresence	SP:C18 column of 150 × 4.6 mm and 3 μm particle size MP: Methanol:acetonitrile:water (30:30:40)	0.2–3 μg/mL	0.015 μg/mL	0.05 μg/mL	85
UPLC	^16^	UV	SP:Waters Acquity UPLC HSS T3, 1.8 µm (2.1 mm × 150 mm, I.D.) column MP:0.1% TFA in water and methanol	0.06–0.18 μg/mL	0.02 μg/mL	–	85
LC	^14^	MS/MS	SP:C18 column MP: Ammonium acetate-acetonitrile (80:20)	0.2–5000 ng/mL	–	0.2 ng/ml	88
LC	^12^	MS/MS	SP:Zorbax Eclipse XDB c,( 4.6 × 150 mm,i.d: *5* μm) MP: Methanol/water/ammonia/formic acid (60:40:0.5:0.01)	0.2–2000 ng/mL	–	0.2 ng/mL	75

*Stationary phase: SP.

*Mobile Phase: MP.

Compared to the previously published methods for the determination of ERD, our work describes four different analytical procedures for the quantitative determination of erdosteine (ERD) as a raw material and in its pharmaceutical dosage form. The suggested methods serve as facile mix and read methods for the simple, rapid, and sensitive determination of ERD while keeping the procedures both ecofriendly and efficient at the same time. Furthermore, this work is the first to present a spectrofluorimetric method for ERD quantitation in bulk and dosage forms. Not only are the suggested methods sensitive and green but also, they are cost effective as they do not require expensive reagents or unaffordable instrumentation.

## Conclusion

This work describes four sensitive green analytical methods for the quantitative determination of erdosteine (ERD) as a raw material and in its pharmaceutical dosage form. Methods I and II rely on spectrophotometric determination of ERD through oxidation by KMnO_4_ and ceric ammonium sulfate, (NH_4_)_4_Ce(SO_4_)_4_.2H_2_O, respectively. On the other hand, in methods III and IV, ERD is determined spectrofluorimetrically using ceric ammonium sulfate, (NH_4_)_4_Ce(SO_4_)_4_.2H_2_O and acriflavine reagents, respectively. Compared to previously reported methods, the developed methods offer many advantages including simplicity, sensitivity and better greenness making them suitable for routine quality control studies. Measurements were recorded directly after simple and rapid procedures involving mixing of few inexpensive, eco-friendly reagents without the need for tiresome analyte extraction and sample pre-treatment steps. Method III comprises the first reported spectrofluorimetric method for ERD and it is the highest sensitive one amongst all proposed methods. Method II and III using ceric comprises the most sensitive spectrophotometric and the highly sensitive pioneer spectrofluorimetric methods, respectively allowing the quantitation of very small concentrations of ERD up to 74 ng/mL. Method I offers the advantages of visible spectrophotometry including high sensitivity in contrast to all reported spectrophotometric methods, least interference from impurities and availability of visible light sources (tungsten lamps or light-emitting diodes) compared to UV sources making it easily accessible for routine laboratory use. The developed methods were validated according to the ICH guidelines and were successfully implemented in the analysis of ERD in its capsules with acceptable accuracy and precision.

### Supplementary Information

Supplementary Information.

## Data Availability

Corresponding author can provide the data upon request.
