# Supplementary material for: Multiple green spectroscopic methods for erdosteine determination in bulk and dosage form with extensive greenness evaluation
Source: Sci Rep. 2023 Oct 25;13:18216. doi: 10.1038/s41598-023-45334-6 (PMC10600230; doi:10.1038/s41598-023-45334-6)
Supplement: Supplementary file 1 — Supplementary Information. [file 41598_2023_45334_MOESM1_ESM.docx]

**Supplementary table S1. Optimum conditions for the determination of ERD using various reagents using the proposed methods.**

| **Condition** | **Method I** | **Method II** | **Method III** | **Method IV** |
| --- | --- | --- | --- | --- |
| **Reagent (I) Volume** | 2 mL  Potassium Permanganate | 1.6 mL  Ceric ammonium sulphate | 1.6 mL  Ceric ammonium sulphate | 2 mL  Acriflavine |
| **Reagent (II) Volume** | 2 mL  Sodium hydroxide | 0.5 mL  Sulphuric acid | 0.5 mL  Sulphuric acid | 1.5 mL  Britton Robinson buffer |
| **Buffer pH** | - | - | - | 5 |
| **Reaction time** | 40 | 40 | 40 | 7 |
| **Temperature** | 25°C | 60°C | 60°C | 25°C |

| **Supplementary figure S1 : Fluoresence Excitation-Emission spectra of the reaction product of 20 μg/mL ERD with acriflavine reagent.** |
| --- |
